# Differentiating Negligent Standards of Care in Diagnosis

**DOI:** 10.1093/medlaw/fwab046

**Published:** 2022-01-12

**Authors:** Kathleen Liddell, Jeffrey M Skopek, Isabelle Le Gallez, Zoë Fritz

**Affiliations:** 1 Faculty of Law, University of Cambridge, Cambridge, UK; 2 (Formerly) Faculty of Law and THIS Institute, University of Cambridge, Cambridge, UK; 3 Addenbrooke’s Hospital, THIS Institute and School of Clinical Medicine, University of Cambridge, Cambridge, UK

**Keywords:** *Bolam*, differential diagnosis, information disclosure, *Montgomery*, negligence, standard of care

## Abstract

Diagnosis lies at the heart of the medical encounter, yet it has received much less attention than treatment. It is widely assumed that negligent diagnosis claims should be governed by the *Bolam* test, but we demonstrate that this is not always the case. First, we disaggregate the diagnostic process into three different acts: forming the diagnosis, communicating it to the patient, and recording it. Second, we consider alternatives to *Bolam* for defining negligence, including less deferential profession-led standards, patient-led standards, and even a reasonable person standard. Third, bringing together these distinctions—within the diagnostic process, and between standards of care—we reveal the unappreciated complexity of negligent diagnosis. Analysing the standard of care that might apply to the three different acts in the diagnostic process, we identify reasons to think that *Montgomery* should apply to the communication of a diagnosis. We also argue that even in areas where the law is well-established, such as the application of *Bolam* to the formation of a diagnosis, challenging questions arise that require further attention. Throughout, the framework and analysis that we develop have significant implications for a set of negligence cases, as well as for medical education, clinical guidelines, and patient care.

## I. INTRODUCTION

In the practice of medicine, the diagnosis of the patient is at the heart of the medical encounter. Without an accurate diagnosis, a doctor cannot appropriately instigate treatment or communicate expected outcomes. Unfortunately, legal academics and practicing lawyers have failed to analyse the diagnostic process with sufficient nuance. 

In order to see the complexities of diagnostic negligence, one must first recognise that diagnosis is not a monolithic act. Rather, as Section II identifies, there are three distinct types of diagnostic acts that should be distinguished: the formation of the diagnosis, the communication of the diagnosis to the patient, and the recording of the diagnosis into the medical file. Further, each of these acts can be performed with respect to the doctor’s diagnosis at different times, as diagnostic uncertainty is gradually eliminated.

When the diagnostic process is unbundled in this way, it becomes clear that a single standard of negligence might not apply to all negligent diagnosis claims—that the deferential test from *Bolam v Friern Hospital Management Committee*^1^ is not the only plausible option. As we explore in Section III, courts could instead adopt a different profession-led standard, a patient-led standard, or even a reasonable person standard.

We then build on these medical and legal distinctions in Section IV, which explores unrecognised complexity in negligent diagnosis. Starting with the question of what standard applies to the formation of a diagnosis, we argue that the applicability of *Bolam* is well established, but that *Muller v King's College Hospital NHS Foundation Trust*^2^ challenges this orthodoxy by drawing a distinction between ‘pure diagnosis’ and ‘pure treatment’ cases. While we ultimately conclude that this distinction is misguided, it does highlight valid concerns about *Bolam*.

Regarding the next phase of the diagnostic process—communication—there is greater uncertainty about the standard of care that applies. Six years ago, *Montgomery v Lanarkshire*^3^ introduced a new patient-led standard of care for the communication of treatment risks and alternatives.^4^ To our knowledge, no courts in England or Wales have explored the application of *Montgomery* to matters of diagnosis, so we look to other jurisdictions for guidance. We analyse a Scottish case^5^ that applies *Bolam*, a Singaporean case^6^ that applies a modified version of *Montgomery*, and US cases that apply a ‘reasonable patient’ standard. We conclude that are good reasons for extending *Montgomery* to include diagnostic information, especially diagnostic uncertainty.

On the question of what standard applies to the recording of a diagnosis, there is very little case law, and we identify reasonable arguments for adopting each of the three standards we have identified (*Bolam*, *Montgomery*, and the reasonable person). We also suggest that the standard might depend, in part, on the foreseeable use of medical records. For example, if the records might be accessed and used by patients following the partnership model of medical care, this could support an argument for the *Montgomery* standard.

It is outside the scope of this article to definitively identify the standard that should apply to all aspects of the diagnostic process. Our aim is to provide a framework that identifies unanswered questions, unexplored possibilities, and an approach to them that will change the outcome in an important set of cases, which we explore below. Of course, our approach will not change the outcome in all cases. This includes cases where the standard of care for diagnosis imposes the same duties as the standard of care that already applies for clinical treatment and management; and cases where a breach of the standard of care for diagnosis does not cause an actionable harm.^7^ Even when our approach does not change the outcome of cases, however, it still matters, since important policy-level implications flow from identifying the standards of care that apply to diagnostic analysis, communication, and recording. These standards need to be incorporated in medical education, clinical guidelines, and patient care.^8^

## II. THE DIAGNOSTIC PROCESS

While diagnosis-related claims are at the core of much litigation against the NHS,^9^ academics and practitioners in the area of medical negligence law often lack a nuanced understanding of the diagnostic process. Unlike in the field of medicine where there are countless textbooks solely dedicated to diagnosis, it is only in the largest practitioner-focused legal hardbacks that the process of diagnosis and the applicable legal principles receive more than a fleeting reference.^10^ In this section, we will cover some key aspects of the diagnostic process and highlight important distinctions that are familiar to doctors but not to lawyers. Subsequent parts of the article demonstrate that these distinctions give us reason to question the assumption that the *Bolam* standard of care applies, and should apply, to all aspects of the diagnostic process.

As doctors know, diagnosis lies at the heart of the medical encounter—and at the heart of medical education. In medical school, students are taught how to take a history and examine a patient, along with identifying potential causes for the symptoms and signs they may encounter. In this way, they learn how to elicit the pertinent information from the patient and match it with an internal database of diagnostic patterns, which are refined through clinical experience. Students are also increasingly being taught about the nature of diagnostic reasoning itself, including the heuristics and biases that might influence their reasoning and decision-making.^11^

From a legal perspective, it is also essential to recognise that the diagnostic process consists of three very different types of actions that have generally been conflated in the literature. The first is the formation of the diagnosis, in which the doctor draws on medical knowledge and the patient’s symptoms and signs to create a list of possible causes for the patient’s presentation. The second is the communication of the diagnosis to the patient and potentially others involved in the patient’s care.^12^ The third is the recording of the diagnosis, in which the medical reasoning (or the output of it) is transferred to the medical records. Unfortunately, these differences have generally been overlooked in the law, which has assumed that negligent diagnosis is a single type of act—and thus that a single legal standard of care, defined by the *Bolam* test, should apply. When the diagnostic process is unbundled in the way we propose, it becomes clear that different standards might apply, as we will explore in more depth below.

Before turning to the law, however, it is important to also highlight that diagnosis is an iterative process in which a doctor whittles down possible explanations for a patient’s condition until uncertainty is gradually eliminated. Doctors distinguish between three different phases of diagnosis: the differential diagnosis (ie, the list of possible explanations for the patient’s signs and symptoms), the working or putative diagnosis (ie, the one that the doctor thinks is most likely and/or needs to be acted on), and the definitive diagnosis (ie, the one established following tests or treatment). The journey from differential to definitive diagnosis is not always linear, however, and definitive diagnosis does not always precede treatment. This is particularly true during the working diagnosis phase, where tentative treatments may be started when time is important (for example, if meningitis is being considered, antibiotics will be given immediately) or to test a response to a particular drug. The process can also backtrack between the phases as new information comes to light. Indeed, a ‘definitive’ diagnosis may never be reached.

In sum, there are two important sets of medical distinctions that have been overlooked in the law. First and most importantly, there are different acts within the diagnostic process (forming, communicating, and recording), which is highly relevant to the law in ways that have gone unrecognised. Second, there is the distinction between the phases of diagnosis (differential, putative, definitive), which due to the doctor’s levels of uncertainty could be relevant to a doctor’s duties regarding any one of the acts.

## III. STANDARDS OF CARE

In order to assess whether a doctor has negligently breached the duty of care owed to a patient, courts must decide what type of standard defines ‘reasonable’ conduct for doctors.^13^ There are potentially three broad approaches that courts might take, each with variations within them.

### A. *The Reasonable Person Standard*

The traditional English and Welsh standard of care in negligence law is the reasonable person standard, as Alderson B stated in 1856:

Negligence is the omission to do something which a reasonable man, guided upon those considerations which ordinarily regulate the conduct of human affairs, would do, or doing something which a prudent and reasonable man would not do.^14^

This reasonable man has also been referred to as ‘the man in the street’^15^ or ‘man on the top of a Clapham omnibus’.^16^ In deciding what a reasonable person would do, courts also take into account the circumstances of the individual.^17^

### B. *Profession-Led Standards*

While the reasonable person standard is applied to a wide and heterogeneous set of cases, courts in the UK and other common law jurisdictions have created an exception in cases involving the exercise of professional skill. For example, in the foundational case of *Bolam*, McNair J explained that when a case

involves the use of some special skill or competence, then the test as to whether there has been negligence or not is not the test of the man on the top of a Clapham omnibus, because he has not got this special skill.^18^

Courts in other jurisdictions have followed the same rationale, but it is important to recognise that there a variety of profession-led standards. In England and Wales, for example, courts follow the ‘*Bolam* test’ applied by McNair J:

The test is the standard of the ordinary skilled man exercising and professing to have that special skill. A man need not possess the highest expert skill; it is well established law that is sufficient if he exercises the ordinary skill of an ordinary competent man exercising that particular art… [He] is not guilty of negligence if he has acted in accordance with a practice accepted as proper by a responsible body of medical men skilled in that particular art.^19^

It is important to recognise that this test incorporates two related but distinct principles, which have been labelled in different ways in the literature. For example, Margaret Brazier and José Miola refer to the ‘two-part test’ created in *Bolam*,^20^ whereas Simon Fox refers to them as two *separate* tests: ‘the skill and care test’ (in the first part of the passage) and ‘the body of doctors test which has become known as “the *Bolam* test”’ (in the second part of the passage).^21^ Rachel Mulheron goes even further in suggesting that ‘the two passages of McNair J’s instruction to the jury address two different legal matters’.^22^ She refers to the first as ‘the *Bolam* standard of care’ and the second as the ‘*Bolam* test of breach’.^23^

Without taking a position on any of these particular approaches, we will use the concept of ‘principles’ to refer to the two broad ways the courts might decide whether a professional was negligent. According to the first principle, the conduct of someone exercising special skill, such as a doctor, should be judged against the conduct of an *ordinary* person *with that special skill*—i.e., the norm for the profession as a whole. According to the second principle, by contrast, the doctor’s conduct does not need to be in line with the norm of the profession as a whole; rather, it need only be in line with a norm accepted by some members. This is significant when there is a difference of opinion within the medical profession, as the judge is not permitted to decide which of the medical opinions is the preferable or more reasonable standard of care.^24^

Both of these principles can also be further refined or modified with exceptions. For example, the law in England and Wales generally adopts the second principle, but a court is able to reject a body of medical opinion ‘if, in a rare case, it can be demonstrated that the professional opinion is not capable of withstanding logical analysis’.^25^ This is known as the ‘*Bolitho* gloss’ after the case of the same name that identified the refinement.^26^ Thus, courts are required to exercise substantial, but not complete, deference to the medical profession. The pros and cons of this approach will be discussed in Section IV.A.3.c. Furthermore, while courts in England and Wales also apply the *Bolam* test to other professions, some commentators argue that the courts apply the test in a particularly deferential way to the medical profession. Lord Woolf, writing extra-judicially, notes that ‘the English courts have at times been less circumspect in reviewing the activities of members of professions other than the medical profession.’^27^ Brazier and Miola adopt a stronger stance and suggest that ‘in other professional negligence claims, time after time, judges have made it clear that expert opinion must be demonstrably responsible and reasonable’, a requirement they consider to be missing in medical litigation.^28^

Finally, it is worth highlighting that courts in other countries have developed profession-led standards that permit much greater scrutiny of medical opinion by adopting the first principle and rejecting the second. For example, in Australia, ‘the standard of care to be observed by a person with some special skill or competence is that of the *ordinary skilled person* exercising and professing to have that special skill’, which is ‘not determined solely or even primarily by reference to the practice followed or supported by a responsible *body of opinion* in the relevant profession’.^29^ Likewise, the law in many states of the US empowers the jury to decide whether a doctor ‘possesses and exercises the degree of skill and learning ordinarily possessed and exercised, under similar circumstances, by other members of their profession.’^30^ Thus, it is certainly possible to have a profession-led standard where the profession is not, in the words of Lord Scarman, ‘a judge in its own cause’.^31^

### C. *Patient-Led Standards*

It is also possible for courts to go beyond mere scrutiny of profession-led standards and replace them with patient-led standards. This is in fact now the norm in information disclosure cases in many common law jurisdictions, where profession-led standards have been rejected on the grounds that medical skill should not dictate what information should be provided to a patient.^32^

For example, in *Montgomery*, the UK Supreme Court held that the standard of care for the disclosure of material risks should no longer be defined by the *Bolam* test.^33^ Adopting a patient-led standard, it held that ‘material risks’ should be disclosed to the patients and further explained:

The test of materiality is whether, in the circumstances of the particular case, a *reasonable person in the patient’s position* would be likely to attach significance to the risk, or the doctor is or should reasonably be aware that the *particular patient* would be likely to attach significance to it.^34^

Note that this test has two limbs, only one of which needs to be satisfied. The first limb is an objective test based on the reasonable patient.^35^ The second limb is a subjective test based on the particular patient in the case, qualified by what the doctor knows or should know.^36^ Thus, under the second limb, a doctor could potentially have a duty to disclose information that would be deemed objectively unreasonable to disclose.^37^ A similar two-limb test has also been adopted in Australia.^38^ In the USA, on the other hand, courts in many states have adopted a patient-led standard that focuses only on ‘the reasonable patient’, not the particular patient.^39^

## IV. NEGLIGENCE IN DIAGNOSIS

In the analysis thus far, we have introduced two important sets of distinctions: between different diagnostic acts, and between different standards of care. In this section, we bring together these distinctions. Exploring the different standards of care that might apply to the formation, communication, and recording of a diagnosis, we reveal the unappreciated complexity of negligent diagnosis.

### A. *Forming a Diagnosis*

#### 1. Profession-Led Standard with Deference

When assessing a claim that a doctor was negligent in forming a diagnosis, courts in England and Wales have traditionally applied the *Bolam* test on the grounds that diagnostic decisions are grounded in clinical judgment.^40^ For example, in the House of Lords case of *Maynard v West Midlands Regional Health Authority*,^41^ the claimant argued that two doctors negligently failed to diagnose tuberculosis promptly. The doctors had thought that the most likely diagnosis was tuberculosis, but that it could be Hodgkin’s disease, which was fatal unless dealt with in its early stages. So they decided to perform a risky exploratory surgery for Hodgkin’s disease, rather than wait several weeks for the results of a tuberculosis test. They performed the surgery non-negligently, but it nevertheless damaged the patient’s left vocal cord. Ultimately a diagnosis of tuberculosis was confirmed. In rejecting the claim of negligent diagnosis, Lord Scarman explained that the doctor’s diagnostic decisions were to be ‘classified as one of clinical judgment’, and therefore *Bolam* applied.^42^ Lord Scarman subsequently affirmed that *Bolam* is the standard required in both diagnosis and treatment cases in the 1985 House of Lords case of *Sidaway.*^43^

While it is clear that the diagnostic decisions in *Maynard* entailed clinical judgment, this is not clear in many of the subsequent cases that have stated that *Bolam* applies to diagnosis. Often, the cases in which these statements appear are not about diagnosis at all; rather, the courts have merely used the phrase ‘treatment and diagnosis’ in cases alleging negligent *treatment*.^44^ In other words, they have assumed without scrutiny that treatment and diagnosis should be governed by the same standard of negligence.

Further, the courts’ elision of diagnosis and treatment matters has received little attention in legal literature, which has failed to explore two possible reasons for treating diagnosis and treatment differently. First, the process of diagnosis might entail duties, such as communication, that do not require technical skills; if so, it is possible that *Bolam* should not apply to all aspects of the diagnosis process. Second, even when diagnosis does entail technical skill, it might not entail matters of clinical judgment; if so, it is possible that courts should not apply the second and more deferential principle of *Bolam* (ie, the instruction to defer to a responsible body of medical opinion). We will return to the first possibility below in Section III.B, and turn now to the second.

The second possibility has been explored in two recent High Court cases: *Muller* and *Brady v Southend University Hospital NHS Foundation Trust.*^45^ In doing so, these cases have resurrected a difference of opinion between the High Court and Court of Appeals in *Penney v East Kent Health Authority*,^46^ which requires some elaboration.

*Penney* concerned three joined cases in which the complainants alleged that their cervical smear tests had been negligently performed by cytoscreeners employed by the defendant hospital. Three important points in the case, somewhat lost in the mists of time, are worth highlighting. First, the experts agreed that the cytoscreeners were trained to observe slides and look for abnormalities, but not to *diagnose* the slides’ clinical meaning like doctors. Second, although the experts’ initial reports disagreed on the obviousness of the abnormalities, they eventually agreed (by the time of the court hearing) that a competent cytoscreener would have seen that it was ‘not a normal slide’.^47^ Third, they agreed that if a screener detected an abnormality or was in any doubt, the screener was trained to pass it on to the senior screener for re-examination. Despite the experts’ agreement on these points, however, the defendant’s two experts stated in court that a competent screener could reasonably have decided the slide was abnormal but benign, and might thus have decided against sending the slide to the senior screener.

In addressing the question of whether *Bolam* should apply to this case, Pepitt J stated *Bolam* was ‘ill-fitting’.^48^ He explained:

I should say at the outset that I find the *Bolam* principle ill-fitting to the facts of Mrs Penney’s case. In *Bolam* and the cases which followed the court was concerned with an aspect of professional conduct of which some members of the profession, but not others, disapproved. In other words, in those cases the defendants’ experts sought to justify as an *acceptable* professional practice what the defendant did or did not do. Here the position is different. All the experts agree that the cytoscreener was wrong. No question of acceptable practice was involved. The issue here was whether the cytoscreeners’ conduct though wrong, was excusable. This seems to me to fall outside the *Bolam* principle… [I]f I am wrong about this I remain of the view that *Bolam* does not assist the defendants. For I do not consider that the evidence of the [experts called by the plaintiffs] stands up to logical scrutiny.^49^

It appears Pepitt J was trying to deal with the twist in the case: namely, that two expert witnesses maintained that cytoscreeners acting competently might not have referred the slide for checking, even though these experts agreed that cytoscreeners were scientists who were not trained to interpret slides and had clear protocols to refer abnormal or potentially abnormal slides. To avoid the implications of these experts’ testimony, Pepitt J concluded that the screeners’ errors should not receive deference under *Bolam*. One way to achieve this would be to abandon *Bolam* completely (his primary rationale); another would be to apply *Bolam* with the *Bolitho* gloss (his alternative rationale).

The case went to the Court of Appeal which rejected Pepitt J’s ‘primary reasoning’,^50^ but upheld his decision under the *Bolitho* gloss. In developing this analysis, Lord Woolf identified three distinct questions, which we think could be useful in deciding other cases as well. He said that the correct approach was to ask:

1. what was to be seen in the slides?
2. at the relevant time, could a screener exercising reasonable care fail to see what was on the slide?
3. could a reasonably competent screener, aware of what a screener exercising reasonable care would observe on the slide, treat the slide as negative?^51^

Lord Woolf explained that the first was a question of fact, so the court needed to make its own finding on the balance of probabilities; the court could be assisted by expert testimony, but *Bolam/Bolitho* did not apply: “[T]he *Bolam* test has no application where what the judge is required to do is make findings of fact. This is so even where those findings of fact are the subject of conflicting expert evidence”.^^52^^ Lord Woolf then explained that the second and third were questions of negligent conduct, so *Bolam/Bolitho* did apply.^53^

Lord Woolf explained that Pepitt J took the correct approach in so far as he held that, on the balance of the probabilities, the slide did in fact show abnormal cells (Question 1); none of the experts argued that the cytoscreener could reasonably have failed to see these abnormal cells (Question 2); and all of the experts agreed at the time of the trial that a reasonably competent cytoscreener would have recognised the abnormality (Question 3). Accordingly, all five experts should have concluded that a reasonable cytoscreener would have referred the slides for re-examination. The two that did not were reasoning illogically and inconsistently, so under *Bolitho*, their body of medical opinion could not support the hospital’s case.^54^

#### 2. Profession-Led Standard Determined by a Judge

While the decision of Lord Woolf and the Court of Appeal in *Penney* is binding law, some lower court judges and academics have since agreed with Peppit J that the deferential principle of *Bolam* should not apply in certain types of diagnosis cases, raising the possibility that *Penney* might be overturned.^55^

The key case challenging the application of *Bolam* is *Muller*. In this case, the central issue was whether Mr Muller’s doctor, Dr Goderya (a consultant histopathologist), was negligent in failing to diagnose cancer on a slide. She received the slide with a small punch biopsy from a wound in his foot and information from his medical history, including that his foot had been injured. After examining the slide, Dr Goderya concluded that the features of the tissue ‘were of an ulcer consistent with a history of trauma’.^56^ Several months later, a larger biopsy taken by another doctor revealed that the patient had a rare malignant skin cancer. At this point, Dr Goderya re-examined the small punch biopsy and agreed that some of the cells may have been cancerous but explained that she misinterpreted them due to other features of the cells.^57^

The expert witness for the hospital testified that Dr Goderya’s misdiagnosis could have been made by a histopathologist acting with reasonable skill and care,^58^ and the hospital’s lawyers argued that the court was required to accept this conclusion unless the *Bolitho* exception applied. Mr Muller’s lawyers disagreed, arguing that the court must ‘determine the objective facts’ about what could be seen on the slides (on which the experts agreed) ‘and then decide for itself whether, in the light of the differing experts views, the misdiagnosis was one that must have been made without the use of reasonable skill and care’.^59^ They argued that the ‘court could not abdicate its responsibility to resolve the conflict of expert opinion by resorting to the *Bolam*-derived notion of a respectable body of medical opinion’.^60^

Faced with the disagreement on whether the deferential ‘body of opinion’ principle of *Bolam* should apply, Kerr J suggested that confusion had arisen ‘because, unfortunately the authorities applying the conventional *Bolam* approach to negligence in this field do not sufficiently differentiate two types of cases’.^61^ He suggested that in the first type of case—which he labelled a ‘pure diagnosis’ case—the condition of the patient is unknown and ‘what is alleged to be negligent is a doctor’s diagnosis of the condition…with no decision made or advice given about treatment or further diagnostic procedures.’^62^ Kerr J concluded that in this type of case, ‘[t]he diagnosis is either right or wrong’, so the court did not need to defer to a body of opinion under *Bolam*.^63^ Kerr J contrasted this with the second type of case, which he labelled a ‘pure treatment’ case, in which ‘the nature of the patient’s condition is known, and the alleged negligence consists in a decision to treat (or advise treatment of) a condition in a particular manner.’ He suggested this type of decision entailed a ‘weighing of risks and benefits’^64^ and that ‘opposed expert opinions may in a sense both be “right”’, which justified ‘particular deference to the views of the experts, whether or not unanimous’.^65^

Applying this distinction to the prior case law, Kerr J explained that *Penney* was a ‘pure diagnosis’ case,^66^*Hunter* and *Bolam* were ‘pure treatment’ cases,^67^ and *Maynard* fell on the spectrum between them but was more akin to a treatment case (as the patient was challenging the doctor’s ‘clinical judgment to perform a risky diagnostic procedure’).^68^ Based on his review of these cases, he concluded:

I have … drawn from it, with some regret, the conclusion that even in a pure diagnosis case such as this, the exercise of preferring one expert to another must be viewed through the prism of the *Bolitho* exception, rather than, as would be preferable, by rejecting the very notion that the *Bolam* principle can apply where no “*Bolam*-appropriate” issue arises. I respectfully agree with Judge Pepitt QC [the trial judge in *Penney*] that the latter approach is more coherent.^69^

However, he noted that in *Penney*, ‘the Court of Appeal did allow a liberal invocation of …[the] *Bolitho* exception’, and he found in Muller’s favour.^70^

In the more recent case of *Brady*, the judge also drew a distinction between ‘treatment cases’ (where there are ‘choices and options available and risks and benefits that need to be considered’) and ‘pure diagnosis’ cases (where ‘there is no weighing of risks against benefits, and no decision to treat or not to treat, just a diagnostic or pre-diagnostic decision, which is either right or wrong’).^71^ But following Kerr J’s analysis *Muller*, which he quoted at length, he agreed that he was required to approach pure diagnosis cases through the lens of *Bolitho*.^72^

#### 3. Which Standard Should Apply?

While the distinction between ‘pure diagnosis’ from ‘pure treatment’ cases might have some intuitive appeal, there are problems with how it is defined in *Muller* and *Brady*, which undermines its ability to justify different legal standards for treatment and diagnosis.

##### a. Matters of Opinion

The core problem is that the distinction between diagnosis and treatment does not, as *Muller* and *Brady* suggest, track the distinction between matters of fact (where there is an objectively right answer, and no judgment) and matters of opinion (where there is no objectively right decision, and judgment is required). This characterisation is incorrect with respect to both diagnosis and treatment decisions.

Starting with ‘pure diagnosis’ decisions, there are three reasons to question the claim that these decisions are right or wrong as a matter of fact. First, much has been written on the sociology of diagnosis^73^ and non-objective ‘facts’^74^ that casts doubt on the view that medical diagnosis is matter of objective fact. It would therefore be surprising if ‘pure diagnosis’, absent judgment, is a clear or well-established category. The problem with this view is nicely illustrated by current debates about the early diagnosis of cancer, as it is generally agreed that there is no clear point at which abnormally dividing cells objectively become ‘cancer’.^75^

Second, even the interpretation of simple diagnostic tests—where apparently little ‘judgment’ is exercised—entails judgments regarding the acceptability of variable sample collections, proxy measurements, ‘normal’ baselines, imperfect assays, the implications of medical history information, and processes of elimination. While some work in medical diagnosis might involve little more than recording and interpreting simple data points (such as blood alcohol levels, pulse, blood glucose levels, blood pressure, etc), even these data points are based on ‘significance thresholds’ which take their cue from population level evidence rather than being tailored to a particular patient. The population-level evidence shelters many assumptions.

Third, a medical professional can interpret a diagnostic test incorrectly as a result of a reasonable judgment call. For instance, reasonable doctors might give different weightings to tests (if the patient has had more than one), the patient’s account of their symptoms, and draw different inferences from the patient’s medical history (possibly incomplete histories). For example, although it may turn out that skin cells are definitely malignant, a competent doctor could conclude from a biopsy that the cells are normal or ‘possibly abnormal’ based on different weightings of features that are unusual (colour, nesting, location), the patient’s medical history (whether they had an injury or another reason for infection), and the possible implications of a false negative or false positive result (such as implications for insurance, anxiety, and risky follow up tests or treatment).^76^ On Kerr J’s typology, a histopathologist making this call is engaged in ‘pure diagnosis’. However, there is considerable judgment and weighing up involved. To give another example, it is difficult to distinguish between sarcoid and sarcoid-mimicking diseases such as primary and secondary cancers, infections, and inflammatory disorders.^77^ Neither a pathologist nor a doctor making this call is engaged in ‘pure’ diagnosis. Many other conditions have mimics that call for judgment when diagnosing. For instance, to diagnose the cause of an interruption of oxygen supply to the brain might involve weighing up evidence to decide whether it was a thrombotic stroke, a bleed, inflammation, or infection.

In these three ways, diagnostic decisions can involve considerably more judgment than is indicated in Kerr J’s definition of ‘pure diagnosis’, but this is not the only problem with the distinction. Cutting the other way, decisions in ‘pure treatment’ cases might, contrary to Kerr J’s suggestion, be right or wrong according to an objective criterion. For example, if it is agreed that a patient should be given the treatment that will maximise their lifespan (an objective criterion), any given treatment will be either right or wrong for that patient. While it might be difficult or impossible to know which treatment will be best, the right treatment is nevertheless a matter of fact. Further, uncertainty about this fact could be resolved by the court based on the balance of probabilities, just like all other factual uncertainties.

##### b. The Weighing of Risks and Benefits

While the fact/value distinction cannot justify treating ‘pure diagnosis’ and ‘pure treatment’ cases differently, *Muller* and *Brady* suggest another reason for distinguishing ‘pure treatment’ decisions: namely, that they involve the ‘weighing of risks and benefits’ whereas ‘pure diagnosis’ decisions do not.^78^

One problem with this is illustrated by our above analysis. For example, it is clear that many (albeit not all) diagnostic decisions also incorporate a weighing of the medical risks and benefits. For example, the doctors in *Maynard* ‘hedged their bets’^79^ and decided to undertake a risky biopsy to rule out another possible diagnosis (Hodgkin’s Lymphoma). Their weighting turned out to be wrong; their initial diagnosis (tuberculosis) was correct, and the biopsy injured the patient. But the court accepted medical evidence that the doctors’ decision to proceed with the biopsy was reasonable, holding that in cases such as this there was room for differences of medical opinion. Kerr J attributed the weighing up of risks in that case to the medical risks associated with biopsy intervention; and distinguished cases like *Muller* involving a diagnostic decision unlinked to medical treatment—so-called ‘pure diagnostic decisions’. However, as discussed above, a case like *Muller* and *Brady* (unlinked to treatment) can involve weighing up risks and benefits such as a false positive or negative affecting insurance, driving, anxiety, employment, quarantine, reproduction, or other life planning.

Another issue with the risk-benefit criterion is that it is not characteristic of all treatments. Just as some diagnostic acts and decisions involve risk-benefit considerations and some do not, the same applies to treatment acts and decisions; some involve careful weighing up, some do not. Simon Fox provides the example of a surgeon who accidentally injures the bowel during routine abdominal surgery.^80^

Thus, in our view, neither this criterion (ie, the involvement of risk-benefit analysis), nor the fact/value distinction, draw a line between diagnosis and treatment decisions. For these reasons, there should not be a general legal difference in how decisions and actions in relation to treatment and diagnosis are governed.

##### c. Reconsidering *Bolam* for Treatment and Diagnosis

Although not persuasive that diagnosis should be governed differently, Pepitt J and Kerr J may have identified a reason for questioning *Bolam* more generally. If a doctor has not made a decision (diagnostic or treatment) based on a weighing of the risks and benefits of the different possible decisions, it is arguable that the highly deferential ‘body of doctors’ principle is not justified. The strong form of *Bolam* deference makes more sense for decisions (treatment or diagnostic) that are formed after weighing the pros and cons of different possible decisions, such that a disagreement emerges within the medical profession about what is in a patient’s best interests.

For years, commentary on *Bolam* has challenged the appropriateness of deferring to a ‘body of doctors’ when a judge concludes, based on expert testimony, that a defendant did not exercise reasonable skill and care.^81^*Bolitho* carved out some circumstances where the judiciary does not defer, but perhaps a wider range of situations should be excepted (such as the situations where the defendant did not weigh up risks and benefits). Or perhaps the *Bolam* principle should be entirely overruled. In place of the *Bolam* principle, the judge could decide, on the basis of evidence, whether the defendant exercised reasonable skill and care. Judges lack medical expertise, but this does not render them incapable of evaluating competing expert testimony and deciding what standard of care is expected by the profession. On the contrary, they make this type of determination in other common law jurisdictions, and they could do so in the UK.

For the time being, however, the current law governing the formation of a diagnosis in England and Wales is Lord Woolf’s three-step approach.^82^ First, the judge must determine what signs and symptoms could be seen and elicited by the doctor at the relevant time, and what the correct diagnosis was. These are factual questions to be resolved by the judge with the benefit of hindsight and on the balance of probabilities, without the application of *Bolam/Bolitho*. Second, there is the question of whether a non-negligent doctor could have failed to recognise the signs and symptoms that indicated the correct diagnosis. Third, there is the question of whether a non-negligent doctor who did recognise the signs and symptoms could have failed to form the correct diagnosis. The second and third questions require the application of *Bolam/Bolitho*.

### B. *Communicating a Diagnosis to the Patient*

Like the legal standard that applies to the formation of a diagnosis, the standard that applies to the communication of diagnostic information has received remarkably little attention in the academic literature and case law. There may be two related reasons for this. First, courts have not recognised the distinction between the communication and formation of diagnosis, treating all diagnostic issues as related to the formation of diagnosis and governed by *Bolam* (as explained above). Second, the courts have routinely focused on information disclosure related to *treatment* (which is the quintessential situation for seeking ‘informed consent’ and the focus of case law), not information related to diagnosis. After *Montgomery*, however, it is possible that courts will recognise the relevance of the first distinction and reject the relevance of the second. In *Montgomery* (a treatment-focused case), the Supreme Court recognised a duty to disclose alternative treatments, if the particular patient or a reasonable patient would consider it relevant to their weighing up of material risks. This would seem to entail a duty to disclose alternative diagnoses that underlie those treatments, plus other treatments (if another diagnosis should turn out to be correct). Further, the principles stated in *Montgomery* could justify a broader duty to disclose diagnostic uncertainty; this might be done out of respect for patient autonomy, or to help the patient decide when to re-seek medical attention. For instance, if a doctor says, ‘come back if it doesn’t resolve’, information about alternative diagnoses could be useful to a generally stoic patient. Unfortunately, there has been little discussion of these possibilities in the post-*Montgomery* case law, so our analysis will focus on cases from Scotland, Singapore, and the USA. We will start with judicial decisions that have adopted a profession-led standard and then turn to those that have adopted a patient-led standard for the disclosure of diagnostic uncertainty.

#### 1. Profession-Led Standard

The only UK case that has directly addressed whether *Montgomery* requires the disclosure of diagnostic uncertainty is the Scottish case of *Taylor v Dailly Health Centre*,^83^ in which the court rejected this suggestion.

In this case, Dr Malloch made a house visit to see Mrs Taylor, who was suffering from pain in her chest and left arm.^84^ Dr Malloch diagnosed her with ‘musculo-skeletal pain and gastro-intestinal upset’ and prescribed pain relief medication.^85^ He did not inform her that his differential diagnosis included Acute Coronary Syndrome (ACS, which includes the possibility of a heart attack).^86^ She died within an hour of his departure.^87^

According to Counsel for the claimant,^88^ Dr Malloch’s failure to disclose this diagnostic uncertainty constituted a failure ‘to obtain Mrs Taylor’s informed consent to the course of action which he decided to take’. As they argued:

He did not inform her that her symptoms and risk factors could mean the presence of ACS and that if she wanted to exclude ACS as a cause, hospital admission by ambulance was required. She had been entitled to be told this information to allow her to make her own assessment. … This failure was a breach of the 2008 General Medical Council Guidelines,^89^ and a breach of the duty incumbent upon him as set out by the Supreme Court in *Montgomery*.^90^

They suggested that if Mrs Taylor had known of her differential diagnosis, she would have acted on this information, attended the hospital, and ultimately survived.^91^ Putting this forward as a legal cause of action, Counsel for the claimant submitted that Dr Malloch failed to obtain Mrs Taylor’s informed consent (per *Montgomery*) to the course of action which he decided to take.

Rejecting this submission, Lord Tyre agreed with the defendant^92^ that *Montgomery* was irrelevant on the grounds that there was an important distinction between: (1) the doctor’s role when considering possible investigatory or treatment options; and (2) the doctor’s role in discussing with the patient any recommended treatment and possible alternatives.^93^ He concluded that the issues that arose in *Taylor* were best placed under the first duty. He explained that the medical decision at issue ‘was a decision falling within the exercise of professional skill and judgment, and not a decision as to which of two or more alternative forms of treatment, carrying differing risks, ought to be undertaken’.^94^ He held that ‘to apply the ratio of *Montgomery* to the circumstances of the present case would … extend it significantly beyond what the Supreme Court’ envisaged.^95^

Lord Tyre went on to dismiss the claimant’s action. He held that Dr Malloch’s diagnosis had been incorrect, but that he had acted in accordance with usual practice for a GP with ordinary skills and competence.

#### 2. Patient-Led Standard

While *Taylor*—the only UK case to address the disclosure of diagnostic uncertainty—held that the issue is governed by *Bolam*, courts in other jurisdictions have departed from *Bolam* and adopted patient-led standards.

One prominent example of this is Singapore, a former British colony where the legal system (including medical negligence law) is based on English common law.^96^ In 2017, Singapore’s Court of Appeal considered whether to follow *Montgomery* in the case of *Hii Chii Kok.* Like the UK Supreme Court, it concluded that *Bolam* should no longer define the duty of disclosure.^97^ However, it went further than *Montgomery*, holding that material information is not ‘limited to risk-related information’ and includes ‘other types of information that may be needed to enable patients to make an informed decision about their health.’^98^ In explaining the types of information that could be material, the Court highlighted information about diagnostic uncertainty:

The factors of certainty and consequence (and context) will necessarily influence what information is reasonably material at every stage. Where the diagnosis is uncertain, more information pertaining to other possible diagnoses will also become material. The pertinent information in this respect may include the degree of certainty, the reasons for the lack of certainty, and whether more can be done to clarify the uncertainty. The possibility of and reasons for a differential diagnosis, if any, will also generally be regarded as material.^99^

Thus, the Court of Appeal ruled that extensive diagnostic information can be material, and its statement that the duty to disclose can arise ‘at every stage’^100^ recognises that the duty does not merely arise in relation to treatment. As Liew and Lijing highlight, the judgment reflects the reality that clinical practice ‘does not rigidly demarcate diagnosis, the provision of advice and treatment’.^101^

The facts of *Hii Chii Kok* centred on the doctors’ finding what *might* have been cancerous neuroendocrine tumours on the patient’s pancreas. An MRI scan was unable to confirm. The doctors conveyed their uncertainty to the patient, alongside the risks of ‘watchful waiting’, and the patient decided to undergo a surgical procedure to remove the lumps. They turned out to be benign, but the patient experienced severe post-operative complications.

While the court ultimately found that Mr Hii’s doctors were not negligent on the grounds that they emphasised the ‘inherent uncertainty’ of the diagnosis and presented him ‘with the option of waiting for six months to repeat the scan … an alternative which stemmed from the uncertainty of the diagnosis’, the case illustrates how the logic of *Montgomery* could be interpreted to require the disclosure of diagnostic uncertainty.^102^

A similar conclusion has been reached by courts in some states in the USA. For example, in the 2012 case of *Jandre v Wisconsin Injured Patients & Families Compensation Fund*,^103^ the Wisconsin Supreme Court held that a physician could have a duty to disclose information regarding diagnostic uncertainty under the ‘reasonable patient’ standard that was in place at the time.^104^ The case concerned a patient who was diagnosed with Bell’s palsy, when he had actually had an ischaemic stroke; he later suffered a ‘full-blown stroke, which impaired his physical and cognitive abilities’.^105^ In suing his doctor for negligence, he made two claims: first, that she was negligent in forming a diagnosis of Bell’s palsy; and second, that she was negligent in failing to inform him that a carotid ultrasound (a non-invasive and readily available diagnostic test) would rule out the possibility that he had suffered a stroke, which she had considered as part of her differential diagnosis for him.

At trial, the jury found that her diagnosis was not negligent, but that her failure to inform was. On appeal, council for defendants argued that the duty to inform only applied after reaching a final diagnosis, not during the diagnostic process:

[W]hen a physician is not negligent in his or her final diagnosis and fully explains to the patient the risks and benefits of treatment alternatives for the condition diagnosed (here, Bell's palsy), the physician has no further obligation to disclose tests or treatments pertaining to other conditions that were included in the physician's differential diagnosis.^106^

But the Court rejected the defendants’ argument, holding that “there are circumstances in which a combination of facts may create a duty … to inform the patient about a diagnostic option that addresses a condition that was eliminated on the way to reaching a non-negligent final diagnosis.”^107^ Thus, in this case, a jury could find that a reasonable person in Jandre’s circumstances would want to know that a carotid ultrasound was available, and that this was necessary to make an informed decision to accept the doctor’s suggestion that he be discharged from the hospital.^108^

#### 3. What Standard Should Apply?

On the normative question of how courts should approach the communication of diagnostic uncertainty to a patient, we think there are two reasons for concluding that diagnostic uncertainty should be disclosed under *Montgomery* (ie, a patient-led standard): the first is preventing harm; the second is respecting autonomy.

##### a. Preventing Harm

There are at least two types of cases in which the disclosure of diagnostic uncertainty could prevent patient harm, and thus be easily justified under the principles articulated in *Montgomery*. The first is when there is fatal or other serious diagnosis on the differential list. The second is when the disclosure would allow the patient to make a more informed decision in response to changing or worsening symptoms.

The potential value of disclosing diagnostic uncertainty in the second type of case—to help patients make more informed decisions about their care—is illustrated by the cases discussed above. For example, the patient in *Jandre* might have sought out a carotid ultrasound and received appropriate surgery which would have prevented his stroke; the patient in *Taylor* might have phoned for an ambulance sooner, preventing her death; and the patient in *Brady* might not have suffered from her delayed diagnosis. Imagine, for instance, that a doctor diagnoses a patient with a condition that is expected to resolve easily with treatment (eg, a chest infection treated with antibiotics), but tells her to return for a follow up test, without explaining that the purpose of the test is to rule out a more serious underlying condition (eg, a tumour) that can only be seen once the first condition is resolved. Without knowledge of the purpose of the follow-up visit, the patient might think that it is not important and post-pone it. During this time, the serious condition could become worse, causing significant harm that could have been avoided if the doctor had explained the diagnostic uncertainty.

Further, this type of disclosure could be significantly more effective than the generic ‘safety netting’ and ‘open door’ language that doctors often use to deal with uncertainty in the diagnostic process. For example, medical students and trainees are often taught to finish patient consultations with an ‘open door’ line such as: ‘If it hasn’t got better by next week, come back and see me again.’ Likewise, they are taught to ‘safety net’ by offering a back-up plan (eg, ‘I think it is indigestion, but if you develop worsening chest pain, call an ambulance’) or a back-up treatment (eg, ‘I don’t think this is a bacterial urinary tract infection, but in case you get a fever or blood in your urine in the next few days, here is a prescription for antibiotics’). However, these approaches can fail, as they are an indirect mode of communication and patients can be unaware that their doctors are trying to put them on ‘alert’ without making them anxious.^109^

##### b. Respecting Autonomy

Another reason for communicating diagnostic uncertainty under a patient-led standard is the principle of respect for patient autonomy recognised in *Montgomery*. As José Miola and Rob Heywood highlight:

If the law relating to information disclosure is about allowing patients to make their own decisions based on all of the relevant information—as *Montgomery* provides—then surely a potential alternative diagnosis, the existence of which may affect the patient’s decision, would be information relevant to the patient’s ability to make the choice that she wants to make.^110^

The Supreme Court already recognised the duty to disclose alternative treatments, and its rationale does not apply only to alternatives that relate to the diagnosis reached by the doctor, but also alternatives that relate to other plausible diagnoses. As the Wisconsin Supreme Court recognised in *Jandre*, there is no good reason for drawing a bright-line distinction between these two types of information.

Providing this information can also enable patients to become more involved in their medical care. While courts have sometimes suggested that patients should be passive in the diagnostic phase of the medical encounter,^111^ the Supreme Court in *Montgomery* emphasised that patients should no longer be treated ‘as the passive recipients of the care of the medical profession’.^112^

Of course, a duty to disclose diagnostic uncertainty could have downsides. First, there is the possibility that patients might be overloaded with information.^113^ Second, there is the possibility that this duty would consume resources (such as doctors’ time and energy),^114^ but produce little benefit—especially considering that most patients struggle with health literacy^115^ and are thus unable to take part in the increasingly complex conversation.^116^ Third, there is the possibility that this requirement would encourage doctors’ to exercise defensive medicine,^117^ and could lead to over-treatment.^118^

Critics of *Montgomery* have long raised the same concerns, but have not yet backed them up with empirical data.^119^ Thus, based on current evidence, we believe that the importance of respecting patient autonomy and protecting them from harm outweigh these apprehensions.^120^

### C. *Recording a Diagnosis*

The importance of medical records is indisputable. They are essential in the care of the patient, acting as a source of constantly evolving information about the patient’s history, diagnosis, treatment, and prognosis, and helping ensure that everyone involved in the patient’s care (which includes the medical team and the patient themselves) has access to all the relevant information. Records can also play an important role in medical litigation. Despite their importance, however, the standard of care applicable to the recording of a diagnosis is not something that the courts or academic community have fully considered.^121^ For this reason, we have more questions than answers in this domain.

#### 1. Profession-Led Standard

In order to find any case law on the standard applicable to the recording of a diagnosis, we had to look beyond the UK to the Canadian case of *Winifredo Gemoto v The Calgary Regional Health Authorit*y.^122^ In this case, where a 7-year-old died following a cardiac arrest,^123^ Martin J found that a doctor was negligent for, amongst other things,^124^ inadequate charting and failing to take and record his vital signs.^125^ The doctor had ‘failed to chart anything after 13:10, including the fact that [the child’s] stomach had become distended at the latest by 16:00’.^126^ In explaining the applicable standard of care, Martin J stated:

[T]he medical practitioner is measured objectively against a reasonable medical person who possesses and exercises the skill, knowledge and judgment of the normal prudent practitioner of his or her special group. In other words, a physician undertakes that she possesses and utilizes the skill, knowledge and judgment of the average reasonable physician.^127^

Thus, Martin J applied a profession-led standard, but her analysis combined both the taking and the recording of the vital signs, so it is not clear whether she would have applied the same profession-led standard to the recording itself.

#### 2. Patient-Led Standard

While we are not aware of any case in which a court applied a patient-led standard to the recording of a diagnosis, this approach might be justifiable in some situations, depending on the potential use of the records.

As traditionally envisioned, the core purpose of medical records is to help doctors and the medical team when treating the patient. As Mathioudakis and others suggest, ‘good clinical record keeping should enable continuity of care and should enhance communication between different healthcare professionals.’^128^ On this account, the records serve not only as an *aide memoir* to the team, but also as a mode of communication between the large and ever-changing medical team involved in treating the patient. This view of the purpose of the medical records is arguably still prominent amongst the medical profession.^129^

This understanding might be challenged, however, by the fact that patients are increasingly aware of their right to access their health records,^130^ and that they are using this right in order to improve their care in various ways.^131^ For example, one empirical study of how patients have used their records concluded:

Record access was used to help prepare patients for consultations, compensate for poor or complex communication during consultations and to reduce the fragmentation of care. Record access had a small impact on health behavior intentions. Overall patients felt that record access reinforced trust and confidence in doctors and helped them feel like partners in healthcare.^132^

The digitisation of the NHS and the adoption of electronic medical records could be expected to increase this type of use.^133^ If so, a court might conclude that the doctor-led standard (for expected medical uses of the record) needs to be supplemented with a patient-led standard (for expected patient uses).

#### 3. The Reasonable Person Standard

It is also conceivable that the recording of a diagnosis could be evaluated under a reasonable person standard following the UK Supreme Court’s decision in *Darnley v Croydon Health Services NHS Trust*.^134^ The case concerned the alleged negligence of an A & E receptionist, and the Supreme Court did not apply *Bolam*, but rather explained that ‘the standard required is that of an averagely competent and well-informed person performing the function of a receptionist at a department providing emergency medical care.’^135^ Thus, there is precedent for using a reasonable person standard in the medical setting. In considering its applicability to the recording of a diagnosis, two questions arise.

The first question is whether the decision to apply the reasonable person standard in *Darnley* was due to the actions being carried out by a receptionist, rather than a medical professional with specialised skills. Perhaps the Court would have applied *Bolam* if the receptionist had been some kind of medical professional, such as a nurse. But this would have been controversial, as *Bolam* should arguably apply only when a medical professional is actually exercising medical skills (or where the patient puts some special trust in their action based on their status as a medical professional). Following this approach, the standard of care for a nurse or receptionist carrying out the work of a receptionist should be the same. On the facts of *Darnley*, the difference between a nurse receptionist and an ordinary receptionist seems to be irrelevant. As Craig Purshouse argues: ‘informing a patient of the expected waiting time for triage does not require any special skill and so the standard of care for clinical receptionists would not be any different to non-clinical ones’.^136^

The same logic could apply to other medical professionals, including doctors, when the conduct at issue does not involve specialised skill. As Lady Hale emphasised in *Montgomery*, the *Bolam* test does not apply to all decisions by medical professionals. Although her statement was focused on situations where doctors make value judgements rather than technical judgements,^137^ there are many circumstances when the actions of a doctor do not rely on special skill (for instance, directing a patient to the car parking area). The idea that *Bolam* is reserved to situations when medical professionals exercise medical skills, rather than applying whenever a person has the status of a medical professional, accords with the origins of *Bolam*. McNair J referred to it as the standard of care applicable to the ‘skilled man exercising and professing to have that special skill’.

The second question, which arises from the first, is whether the act of recording a diagnosis requires special skill—and here, it seems that the answer depends on how one frames the activity of recording a diagnosis. For example, if one conceptualises a diagnosis as the end-point of medical reasoning, it seems there is a good argument that the reasonable person standard should apply to the recording of it. The claim would be that once the doctor has reached the diagnosis, she can dictate it to an assistant with no medical training to record; and thus, even if she records it herself, there is no medical skill involved. However, one could also conceptualise the recording process more broadly as including some medical reasoning. For example, one might argue that the recording includes the process by which the doctor synthesises complex information and decides what should go in the record and be prioritised. On this account, the recording clearly involves medical skill; some have even argued that ‘medical record keeping has evolved into a science of itself’.^138^

#### 4. What Standard Should Apply?

It would be premature to put forward a view as to the standard of care that should apply for the recording of a diagnosis. As explained, there are robust arguments for three different options: a professional-led standard if medical skill is exercised in recording a diagnosis; a patient-led standard if patients are relying on their medical records to engage with their own care; and a reasonable person standard if recording a diagnosis involves no special professional skills.

To evaluate this further, it would be useful to investigate the recording process and how doctors and other medical professionals input their medical records. Key questions include ascertaining what level of detail is usually provided in records,^139^ particularly when inputting a differential diagnosis. It would also be useful to research the extent to which patients are accessing and using medical records to support their understanding and decision-making.

There will be situations where liability for negligent recording and the applicable standard of care could make a significant difference to a patient. For example, imagine that someone with chest pains and concerns about a heart attack goes to a hospital where a doctor diagnoses him with ‘costochondritis’, an inflammation where the ribs meet the sternum. The doctor explains that there is some uncertainty in this diagnosis, but that the tests needed to rule out the alternative diagnosis of angina are not recommended at present due to their risks. The doctor tells the patient to return to the hospital for these tests if his symptoms worsen during exercise. However, the doctor only records the diagnosis of ‘costochondritis’ in the hospital records and in the letter to the patient’s GP. The doctor does not record the diagnostic uncertainty, nor the differential diagnosis of angina. When the patient later returns to his GP, he has forgotten about the possibility of angina, and asks for pain relief for his ‘costochondritis’ which he says has worsened. The GP prescribes it without considering the differential, nor the need for further tests. Two weeks later, the patient has a heart attack.

Stipulating that the hospital doctor was not negligent in forming her diagnosis or communicating with the patient about it, her failure to record the differential could still be negligent and the applicable standard could be crucial in deciding this.^140^ If *Bolam* applies, the doctor might avoid liability merely by demonstrating that other doctors would not have recorded the alternative diagnosis. If *Montgomery* applies, however, the patient could argue that the doctor was negligent on the grounds that the alternative diagnosis was material, and that if it had been recorded, he would have returned to the hospital when his pain worsened. Finally, if the reasonable person test applies, the key question might be whether, having identified and discussed the possible alternative diagnosis, the doctor acted reasonably in not writing it in the record—a question one could imagine being decided either way.

Finally, it is worth highlighting that as with formation and communication, resolving the standard for the recording of diagnoses should inform medical education, clinical guidelines, and the design of health records software (which should, for example, have appropriate text boxes and prompts).

## V. CONCLUSION

This piece has shown that courts and legal scholars have failed to recognise the complexity of negligent diagnosis. They have overlooked important differences between the formation, communication, and recording of a diagnosis, and thus assumed that one standard of care—defined by the *Bolam* test—applies to all negligent diagnosis claims.

While some might argue that our call for differentiation of the diagnostic process will overly complicate medical negligence claims and be too burdensome on doctors, *Montgomery* demonstrates that the mere fact that it would be simpler to apply one standard to all negligence claims is not an adequate justification for doing so.^141^

The application of *Bolam* to the formation of a diagnosis is well established, and we have argued that there should not be different treatment of ‘pure diagnosis’ and ‘pure treatment’. Nevertheless, the strong form of *Bolam* deference might be unjustified in some cases of diagnosis and treatment and the courts may need to consider this in the future. In the event that medical opinions differ, perhaps a judge should only defer to the standard accepted as reasonable by the defendant’s experts where the case involves a weighing up of information involving medical skill and training, or perhaps only where it involves a weighing up of pros and cons for the patient’s interests. Regarding the standard of care that should be applied to communication, which is not well established in case law, we have argued that the application of *Montgomery* could be justified on two different grounds: prevention of harm and respect for patient autonomy. Finally, we have suggested that the recording of a diagnosis could plausibly be judged under *Bolam*, *Montgomery*, or a reasonable person standard, depending on the nature of the record and the context.

We want to highlight, however, that the overall aim of this article is not to definitively identify the standard that should apply to all aspects of the diagnostic process, but rather to provide a framework that helps identify the unanswered questions and unexplored possibilities. As our analysis reveals, the diagnostic process and the standards of care that apply to it warrant much more attention by academics and the courts. For example, empirical research about how diagnosis, communication, and recording take place in practice would be useful, as would further research on the principles by which cases involving diagnosis are settled out of court. This is needed not only to improve the normative foundations of the law, but also to provide doctors and patients with greater clarity about their rights and duties.

## Footnotes

1 [1957] 1 WLR 582 (QB).

2 [2017] EWHC 128 (QB).

3 [2015] UKSC 11 (SC).

4 I Le Gallez and others, *Montgomery*’s Legal and Practical Impact: A Systematic Review at 6 Years (2021) Journal of Evaluation in Clinical Practice, doi: 10.1111/jep.13620. Online ahead of print.

5
*Taylor v Dailly Health Centre and others* [2018] CSOH 91.

6
*Hii Chii Kok v (1) Ooi Peng Jin London Lucien; (2) National Cancer Centre* [2017] SGCA 38.

7 For example, a loss of autonomy is not currently recognised as actionable harm—though this could change.

8 Our legal analysis forms one part of a broader multi-disciplinary project that seeks to unpack the complexity of the diagnostic process in order to improve how differential diagnosis is made, communicated and recorded in the acute care setting. For more see https://www.thisinstitute.cam.ac.uk/research-projects/improving-how-differential-diagnosis-is-made-communicated-and-recorded-in-acute-care/.

9 NHS Resolution data suggest that diagnosis-related claims comprise one of the largest categories of cases, second only to treatment-related claims. NHS Resolution, FOI_4291, 10 March 2020.

10 See M Jones, *Medical Negligence* (5th edn, Sweet & Maxwell 2018) [4-10]–[4-50]; J Laing and others (eds) *Principles of Medical Law* (4th edn, OUP 2017) s 4.74.

11 Heuristics are ‘rules of thumb’ or shortcuts which people use in decision-making. They can be useful, but also can shut down objective reasoning, and are susceptible to biases, including availability (what has been recently experienced), representativeness (how representative the sample witnessed is of the general population), and anchoring (the first experiences) biases. For more see A Tversky and D Kahneman, ‘Judgment under Uncertainty: Heuristics and Biases’ (1974) 185(4157) Science 1124; JL Bowen, ‘Educational Strategies to Promote Clinical Diagnostic Reasoning’ (2006) 355(21) New England Journal of Medicine 2217; JP Kassirer, ‘Teaching Clinical Reasoning: Case-based and Coached’ (2010) 85(7) Academic Medicine 1118; DP Sklar, ‘Teaching the Diagnostic Process as a Model to Improve Medical Education’ (2017) 92(1) Academic Medicine 1.

12 We will only be examining the communication with the patient.

13 For those unfamiliar with a negligence claim in the UK, there are three parts needed for a successful claim. The claimant must establish: first, that the defendant owed them a duty of care; second, that the defendant breached this duty; and third, that the breach caused the claimant to suffer actionable damage.

14
*Blyth v Birmingham Waterworks* (1856) 11 Ex 781 (Exchequer), [784].

15
*Bolam* (n 1) [586].

16
*Hall v Brooklands Auto Racing Club* [1933] 1 KB 205 (KB), [224].

17 C Walton and others, *Charlesworth & Percy on Negligence* (14th Consolidated mainwork incorporating second supplement 2020) 8-06.

18
*Bolam* (n 1) [586].

19 ibid [586]–[587].

20 M Brazier and J Miola, ‘Bye-bye *Bolam*: A Medical Litigation Revolution?’ (2000) 8(1) Medical Law Review 85, 87.

21 S Fox, ‘*Bolam* Is Dead. Long live *Bolam*!’ (2019) 4 Journal of Personal Injury Litigation 213.

22 R Mulheron, *Principles of Tort Law* (2nd edn, CUP 2020) 7.11.

23 ibid.

24
*Maynard v West Midlands Regional Health Authority* [1984] 1 WLR 634 (HL).

25
*Bolitho v City & Hackney Health Authority* [1998] AC 232 (HL), [243].

26 ibid.

27 H Woolf, ‘Are the Courts Excessively Deferential to the Medical Profession?’ (2001) 9(1) Medical Law Review 1.

28 Brazier and Miola (n 20) 88.

29
*Rogers v Whitaker* (1992) 109 ALR 625, [631]. The Court further explained that ‘[e]ven in the sphere of diagnosis and treatment, the heartland of the skilled medical practitioner, the Bolam principle has not always been applied’, ibid, citing *Albrighton v Royal Prince Alfred Hospital* [1980] 2 NSWLR 542 and *E v Australian Red Cross Society* (1991) 99 ALR 601.

30 BJ Van Arsdale and JK Levin, ‘§ 105 VI. Malpractice, Negligence and Other Wrongful Acts B. Degree of Care and Skill’ Corpus Juris Secundum September 2020 Update 70.

31
*Sidaway v Board of Governors of the Bethlem Royal Hospital* [1985] AC 871 (HL), [882].

32
*Rogers v Whitaker* (n 31) [625]: ‘no special medical skill is involved in disclosing the information’.

33 See *Sidaway* (n 33).

34
*Montgomery* (n 3) [87] italics added.

35 This approach was adopted pre-*Montgomery* in *Pearce v United Bristol Healthcare NHS Trust* [1999] ECC 167, [21]: ‘if there is a significant risk which would affect the judgment of a reasonable patient, then in the normal course it is the responsibility of a doctor to inform the patient of that significant risk, if the information in needed so that the patient can determine for him or herself as to what course he or she should adopt.’

36 This was the standard used in *Rogers v Whitaker* (n 31).

37 See T Elliott, ‘A Break with the Past? Or More of the Same?’ (2015) 31(3) Professional Negligence 190, 193 drawing upon M Brazier, ‘Patient Autonomy and Consent to Treatment: The Role of the Law?’ (1987) 7(2) Legal Studies 169, 175: ‘a patient with cancer of the throat may choose to undergo radiation over the more effective option of surgery, on the basis that they would prefer to live with the power of normal speech.’

38
*Rogers v Whitaker* (n 31) [634]: ‘a risk is material if, in the circumstances of the particular case, a reasonable person in the patient's position, if warned of the risk, would be likely to attach significance to it or if the medical practitioner is or should reasonably be aware that the particular patient, if warned of the risk, would be likely to attach significance to it.’

39 RF Wilson, ‘The Promise of Informed Consent’ in IG Cohen, AK Hoffman and WM Sage (eds), *The Oxford Handbook of US Health Law* (OUP 2017) 218.

40 See *Tracey Bell v Bedford Hospital NHS Trust* [2019] EWHC 2704 (QB), [35]; *Daniel Meynell v Croydon Health Services NHS Trust* [2018] EWHC 2431 (QB), [94] and *Mrs Julia Coakley v Dr Henrietta Rosie* [2014] EWHC 1790 (QB), [59].

41
*Maynard* (n 25).

42 ibid [638].

43
*Sidaway* (n 33) [881]–[882].

44 See, eg, *Hunter v Hanley* [1955] SC 200 (COS, IH, FD), [204].

45 [2020] EWHC 158 (QB).

46 [1999] Lloyd’s Rep Med 123 (QB); [2000] PNLR 323 (CA).

47
*Penney* (CA) (n 48) [333].

48
*Penney* (QB) (n 48) [130].

49 ibid.

50
*Brady* (n 47) [25].

51
*Penney* (CA) (n 48) [331].

52 ibid.

53 ibid.

54 ibid [335]–[336].

55 See *Muller* (n 2); *Brady* (n 47); Fox (n 21).

56
*Muller* (n 2) [14].

57 ibid [31].

58 ibid [42]–[44].

59 ibid [46]–[47].

60 ibid [47].

61 ibid [48].

62 ibid [48], [62].

63 ibid [49].

64 ibid [60] referring to *Bolitho* (n 26) [242].

65
*Muller* (n 2) [50].

66 ibid [62].

67 ibid [55].

68 ibid [58]–[61].

69 ibid [72]–[73].

70 ibid [72].

71
*Brady* (n 47) [23]. In this case, the plaintiff experienced acute pain several months after an appendectomy. She saw several doctors, whose differential diagnosis included omental infarction, infection or a malignancy. She was told that it was probably an infarction that would resolve with antibiotics. But after six weeks, she returned to the hospital in continued pain. While her doctor said that a gastroscopy was required to achieve a definitive diagnosis, she failed attend her scheduled appointment—and her rescheduled appointment, as she was feeling better. However, around 3 months after the gastroscopy was first ordered, she returned to the hospital in pain. She she was diagnosed with a serious infection, requiring surgery under general anaesthetic to drain the infected site. She sued her doctors for negligence in forming their original misdiagnosis. ibid [1]-[17].

72 ibid [27]. For more on *Brady,* see SA Schnobel, ‘*Brady v Southend University Hospital NHS Foundation Trust* [2020] EWHC 158: ‘Pure Diagnosis’ Claims and Setting the Professional Standard of Care’ (2021) Medical Law Review fwaa040 <https://doi.org/10.1093/medlaw/fwaa040> accessed 5 May 2021.

73 See, eg, A Jutel and P Conrad, *Putting a Name to it: Diagnosis in Contemporary Society* (Johns Hopkins University Press 2011); A Jutel, ‘Diagnosis: A Critical Social Reflection in the Genomic Era’ (2019) 24 Ciência & Saúde Coletiva 3619.

74 See J Reiss and S Jan, ‘Scientific Objectivity’ (Stanford Encylopedia of Philosophy, 30 October 2020) <https://plato.stanford.edu/entries/scientific-objectivity/> accessed 5 May 2021.

75 It is also important to remember that many medical *conditions* are *defined* by judgement rather than fact, such that judgement is ‘baked’ into the diagnosis and could be challenged by some doctors. This is obvious for conditions such as depression, autism, ADHD, Crohn’s disease, syndromic diseases, psychiatric disorders. It also occurs in radiology and histopathology.

76 For example, stroke is defined as an abrupt onset of brain malfunction due to interruption of the blood supply, but there are many other conditions that can have the same result (eg seizure, sepsis, brain tumour), a diagnosis of stroke rather than sepsis could have major implications for the patient’s insurance and driving licence. See P Hand and others, ‘Distinguishing Between Stroke and Mimic at the Bedside’ (2006) 37(3) Stroke 769.

77 A Jameson and others, ‘Sarcoidosis, the Master Mimicker’ (2020) Current Problems in Diagnostic Radiology <https://doi.org/10.1067/j.cpradiol.2020.10.013> accessed 30 November 2020.

78
*Muller* (n 2) [48], [62]; *Brady* (n 47) [23].

79
*Muller* (n 2) [55].

80 Fox (n 21) 213.

81 See, eg, JP Swoboda, ‘*Bolam*: Going Going…Gone’ (2018) 1 Journal of Personal Injury Litigation 9; Brazier and Miola (n 20).

82
*Penney* (CA) (n 48) [331].

83
*Taylor* (n 5).

84 ibid [2].

85 ibid.

86 ibid.

87 The cause of death was listed as coronary artery atherosclerosis which is linked to the differential diagnosis of ACS.

88 We use the term claimant for consistency despite the Scottish legal system adopting the term pursuer.

89 The General Medical Council, ‘Consent: Patients and Doctors Making Decisions Together’ 2008. The relevant section of the guidance is section nine which stipulates that doctors must give patients information they want or need about: (i) the diagnosis and prognosis, (ii) any uncertainties about the diagnosis or prognosis, including options for further investigations. This section was reproduced in the recently released 2020 update of the Consent guidance, which also includes a separate section exclusively dedicated to advising doctors on how to deal with uncertainty.

90
*Taylor* (n 5) [36].

91 ibid.

92 We use the term defendant for consistency despite the Scottish legal system adopting the term defender.

93
*Taylor* (n 5) [40].

94 ibid [48].

95 ibid.

96 Pre-*Montgomery* the Singaporean courts were mirroring the UK debates concerning whether *Bolam* should govern information disclosure cases. See *James v Gunapathy D/O Muniandy* [2002] SGCA 25.

97
*Hii Chii Kok* (n 6) [2].

98 ibid [138].

99 ibid [143] italics omitted.

100 ibid.

101 C Liew and T Lijing, ‘*Montgomery* and *Bolitho:* Is there a Practical Difference?’ (2017) October Singapore Medical Association 18, 19.

102
*Hii Chii Kok* (n 6) [193].

103
*Jandre v Wisconsin Injured Patients & Families Compensation Fund* 813 NW2d 627 (Wis 2012).

104 Following legislative amendment in 2013, this is no longer the law in Wisconsin. The relevant state statute now adopts a ‘reasonable physician’ standard and specifies that the physician is not required to disclose ‘[i]nformation about alternate medical modes of treatment for any condition the physician has not included in his or her diagnosis at the time the physician informs the patient’. Wisconsin Statutes Annotated s 448.30 (published 15 April 2021).

105
*Jandre* (n 105) [641].

106 ibid [644].

107 ibid [660].

108 ibid [656].

109 See KL Rising and others, ‘Emergency Medicine Resident Perceptions About the Need for Increased Training in Communicating Diagnostic Uncertainty’ (2018) 10(1) Cureus e2088.

110 R Heywood and J Miola, ‘The Changing Face of Pre-operative Medical Disclosure: Placing the Patient at the Heart of the Matter’ (2017) 133 Law Quarterly Review 296, 309.

111 For example, in the Singaporean case of *Noor Azlin Bte Rahman v Changi General Hospital Pte Ltd* [2018] SGHC 35, [54] Judge Belinda Ang Saw Ean stated that patient should play an active role in decision-making about treatments and procedures, but that ‘when a patient is being diagnosed and treated, her role is largely passive.’

112
*Montgomery* (n 3) [75].

113 G Murphy, ‘Championing Autonomy—Does *Montgomery* Truly Protect Patient's Interests’ (2018) 6 North East Law Review 35, 41.

114 MD Ginsberg, ‘Informed Consent and the Differential Diagnosis: How the Law Can Overestimate Patient Autonomy and Compromise Health Care’ (2014) 60(2) Wayne Law Review 349, 394.

115 See S Kyle and D Shaw, ‘Doctor-Patient Communication, Patient Knowledge and Health literacy: How Difficult Can It All Be?’ (2014) 96(6) The Bulletin of the Royal College of Surgeons of England e9.

116 Ginsberg (n 117) 393.

117 ibid 392.

118 ibid 391.

119 There are however, a few empirical pieces on doctor’s awareness of *Montgomery.* Eg, SR Knight and others, ‘Patient Consent in the Post-*Montgomery* Era: A National Multi-speciality Prospective Study’ (2019) 17(5) The Surgeon 277; C McKinnon and others, ‘Surgical Consent Practice in the UK following the *Montgomery* Ruling: A National Cross-sectional Questionnaire Study’ (2018) 55 International Journal of Surgery 66.

120 For more on this issue, see E Cave and C Milo, ‘Informing Patients: The *Bolam* Legacy’ (2020) 20(2) Medical Law International 103.

121 This is not to say that all aspects of medical records have been neglected. On the contrary, the legal literature and case law have explored the purpose of medical records, their utility in future litigation and confidentiality concerns.

122 2006 ABQB 740, 2007 2 WWR 243.

123 ibid [1].

124 The negligence focused on the inadequacies in care, monitoring, assessment, and treatment.

125
*Winifredo* (n 125) [55].

126 ibid [46].

127 ibid [50].

128 A Mathioudakis and others, ‘How to Keep Good Clinical Records’ (2016) 12 Breathe 371.

129 A Crucefix and others, ‘Sharing a Written Medical Summary with Patients on the Post-Admission Ward Round: A Qualitative Study of Clinician and Patient Experience’ (2021) 27(6) Journal of Evaluation in Clinical Practice 1235 .

130 See, for example, Access to Medical Reports Act 1988 c28; Access to Health Records Act 1990 c23; Data Protection Act 2018 c12. In the USA, the 21st Century Cures Act requires that the Secretary of Health and Human Services ‘use existing authorities to encourage partnerships … with the goal of offering patients access to their electronic health information in a single, longitudinal format that is easy to understand, secure, and may be updated automatically.’ 21st Century Cures Act 2016 s 4006.

131 B Fisher, V Bhavnani and M Winfield, ‘How Patients Use Access to their Full Health Records: A Qualitative Study of Patients in General Practice’ (2009) 102(12) Journal of the Royal Society of Medicine 539.

132 ibid.

133 NHS England, Digital Transformation <https://www.england.nhs.uk/digitaltechnology/> accessed 5 May 2021.

134 [2018] UKSC 50 (SC).

135 ibid [25].

136 C Purshouse, ‘The Impatient Patient and the Unreceptive Receptionist: *Darnley v Croydon Health Services NHS Trust* [2018] UKSC 50’ (2019) 27(2) Medical Law Review 318, 324.

137
*Montgomery* (n 3) [115].

138 J Thomas, ‘Medical Records and Issues in Negligence’ (2009) 25(3) Indian Journal of Urology 384; SA Nouraei and others, ‘A Study of Clinical Coding Accuracy in Surgery: Implications for the Use of Administrative Big Data for Outcomes Management’ (2015) 261(6) Annals of Surgery 1096.

139 P Jones, T Hughes and T Morton, ‘Information Technology Coding Systems in the Emergency Department: It Is Not the Tools, It Is How We Use Them’ (2019) 31(5) Emergency Medicine Australasia 700.

140 The GP’s failure to re-evaluate the patient and the diagnosis could also be negligent, but we are focusing here on the potential negligence of the hospital doctor.

141 J Badenoch, ‘Montgomery and Patient Consent: Perceived Problems Addressed’ (2016) 22(1–2) Clinical Risk 12, 14.

